# Malignant leydig cell tumor in a 91-year-old man: Case report

**DOI:** 10.1590/S1677-5538.IBJU.2018.0720

**Published:** 2019-12-17

**Authors:** Roberto Mateussi Justo, Elizeu Bernabé, Millian Carlos Ronchini, Eclair Lucas, Gilberto Saber, Luís Cesar Záccaro da Silva

**Affiliations:** 1 Departamento de Urologia da Santa Casa de Misericordia de Ribeirão Preto, Sao Paulo, Brasil

**Keywords:** Leydig Cell Tumor, Testis, Neoplasms

## Abstract

Testicle tumors are a rare entity among men population, accounting for only 1-1.5% of all cancers among men. The stromal tumors of the sexual cord correspond just 4% of all testicular cancers. Only 10% of them are malignant. The major representative of the sex cord-stromal tumors is the Leydig cell tumor, corresponding to 75 to 80% of the total. It has bimodal age incidence, involving children and adults between 30 and 60 years. We report the caso of a 91-year-old man with malignant Leydig cell tumor, presenting increase of the volume of scrotum, local pain and hyperemia. The are few cases in the literature, only 1 with pacient above 85 years old.

## INTRODUCTION

Testicular neoplasms are a rare entity among men, accounting for only 1-1.5% of all male cancers. Most tumors are germinal (seminoma and nonseminoma).). The incidence of germ-cell tumors (GCTs) decreases after 50 years of age, when the incidence of spermatocytic seminoma, primary lymphoma, stromal tumors and metastasis increases. IN ADDITION, the incidence of GCTs in the elderly population is extremely rare, with the exception of spermatocytic seminomas, a distinct GCT, with a generally benign behavior (1).

Stromal testicular neoplasms correspond to just 4% of all testicular cancers. Only 10% of them are malignant. Sex cord-stromal tumors can be divided into: Sertoli cells, Leydig cells, granulosa cells and theca cells (2).

## CASE REPORT

A 91 years old man presented an increase of the volume of the scrotum for approximately 1 year, with local pain and hyperemia for 7 months. He sought medical attention at the time and was treated with antibiotic therapy for epididymo-orchitis. When the symptoms persisted, he was referred to the Urology outpatient clinic of the Santa Casa de Misericórdia de Ribeirão Preto.

At the physical examination, he presented with an enlarged scrotum on the left with transillumination showing fluid, without hyperemia. In the consultation, a scrotal sonogram was requested.

On the return, he presented an ultrasound report of hydrocele with fine debris in the left side, with a nodular, solid, rounded, partially defined, hypoechoic image with increased flow to the Doppler study measuring 2.0 x 1.4 x 1.1cm. The patient complained of dysuria and polyuria, and we opted for treatment with antibiotic guided by urine culture and surgical treatment afterwards.

A frontal chest radiography was performed as a first imaging procedure: it showed diffuse osteopenia and ectasia of the aorta. Computed tomography (CT) imaging revealed left renal cyst and infra-centimetric bilateral inguinal lymph nodes.

After 3 months, a left unilateral orchiectomy was performed via inguinal, with hydrocele correction. Material was sent for histopathology.

Macroscopic examination revealed left testis measuring 5.8 x 2.9 x 2.7cm, with a smooth outer surface and cut with a yellowish, spongy parenchyma, containing a brown nodule, firm, well delimited and homogeneous, measuring 1.7 x 1.5cm, restricted to the parenchyma. Microscopy showed neoplasia consisting of cells with a hypertrophic nucleus, sometimes with evident nucleolus and broad and eosinophilic cytoplasm, all contained in the testicular parenchyma, with no evidence of infiltration in testicular coating. Absence of invasion of vein and lymphatics. Epididymis and spermatic cord without evidence of neoplastic infiltration. Margin of surgical resection of the spermatic cord free of neoplasia. Pathologic staging: pT1, pNx, pMx. Immunohistochemical exam was positive for inhibin, calretinin, melan-A and KI-67. Diagnosis was compatible with Leydig cell tumor.

He returned to the outpatient clinic after 1 month of surgery, with the presence of hematoma in scrotum, confirmed by scrotal sonogram. We opted for a conservative treatment.

## DISCUSSION

The major representative of the stromal tumors is the Leydig cell tumor. It corresponds to 75 to 80% of all cases. There is no association with cryptorchidism. It has bimodal age incidence, involving children and adults between 30 and 60 years. Children account for 25% of cases. Elderly people tend to have malignant tumors (2).

The first article to describe the ultrastructure of a Leydig's tumor, which appeared in a 3-year-7-months-old boy, was by Cervos-Navarro and associates in 1964 (3). It is a rare tumor with few citations in articles. G. Cruceyra Betriu et al. reported 8 cases in one review, during the period from 1985 to 2000, with a median age of 33.5 years, ranging from 8 to 60 years (4). Another review by Luca Carmignani uring the period from 1990 to 2004 operated on 24 patients aged 22-61 years at three centers (5).

There are few cases of this histological type in patients over 80 years of age (6). Here we report a case of a patient with this histological type and aged over 90 years, which evidences the need to think about differential diagnoses of scrotal masses in the elderly (Table-1). Primary lymphoma is an uncommon disease that comprises only 1-9% of testicular neoplasms. However, it is the most common malignancy in men in this age range and 85% of cases are diagnosed in men older than 60 years old.

**Table 1:** Scrotal masses in elderly men.

TESTICULAR
Primary Lymphoma
Stromal Tumors
Spermatocitic Seminoma
Metastasis
Epidermoid Cyst
Leydig Cell Hyperplasia
Fibroma Of Gonadal Origin
Hemangioma
PARATESTICULAR
Lipomas
Adenomatoid Tumors
Leiomyomas
Testicular Appendage With Torsion
Fibrous Pseudotumor
Liposarcoma
Leiomyosarcoma
NONTUMOROUS CONDITIONS
Testicular Infarction
Hematoma
Orchitis
Granulomatous Diseases (Eg, Sarcoidosis And Tuberculosis)

In addition, about 2-3% of these tumors are extratesticular and arise from paratesticular tissue. The paratesticular region comprises: the spermatic cord, testicular tunics, epididymis, and vestigial remnants. Although uncommon, these tumors have been recorded as the main urogenital location for sarcomas in the elderly (7).

Approximately 90% of the testicular masses are benign and only 10% are malignant. Features of malignancy include larger tumors (>5cm), infiltrative margins, foci of necrosis, angiolymphatic invasion, nuclear atypia, mitotic count >3/10 hpf, DNA aneuploidy, and increased MIB-1 activity (8). Although the most reliable criterion is the presence of metastasis (9).

Adults usually present with painless testicular masses, orchialgia, gynecomastia, impotence, decreased libido or infertility. Children are usually aged between 5 and 10 years old and present with testicular masses and precocious signs of virilization, including pubic hair, increased penis size and acne due to abnormal amounts of testosterone. In this age group should be made differential diagnosis with causes of early puberty such as: congenital adrenal hyperplasia, adrenocortical carcinoma and isosexual precocious puberty (2).

**Figure 1:**
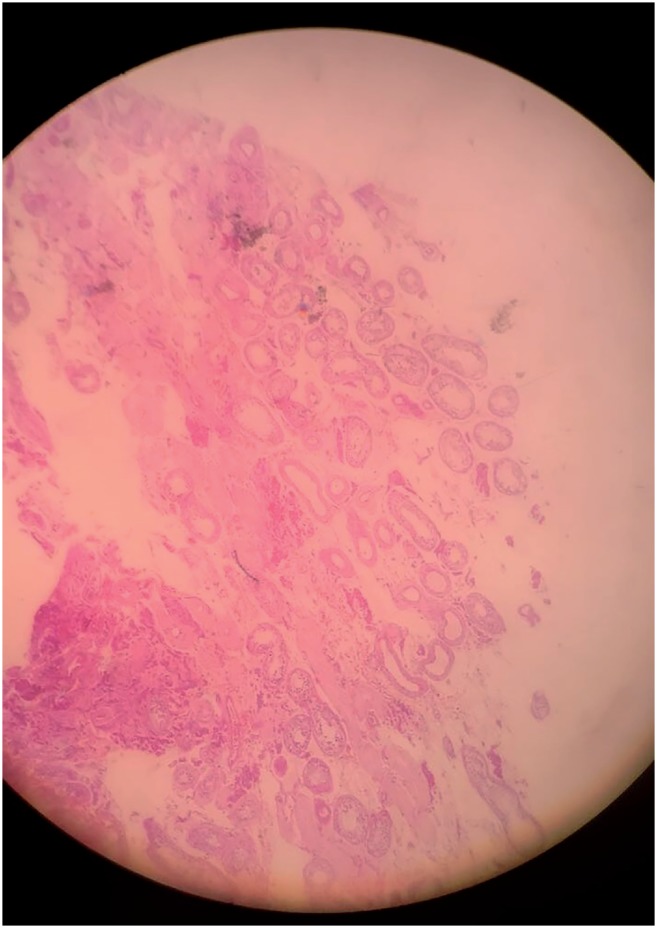
Epididymis (4x).

**Figure 2:**
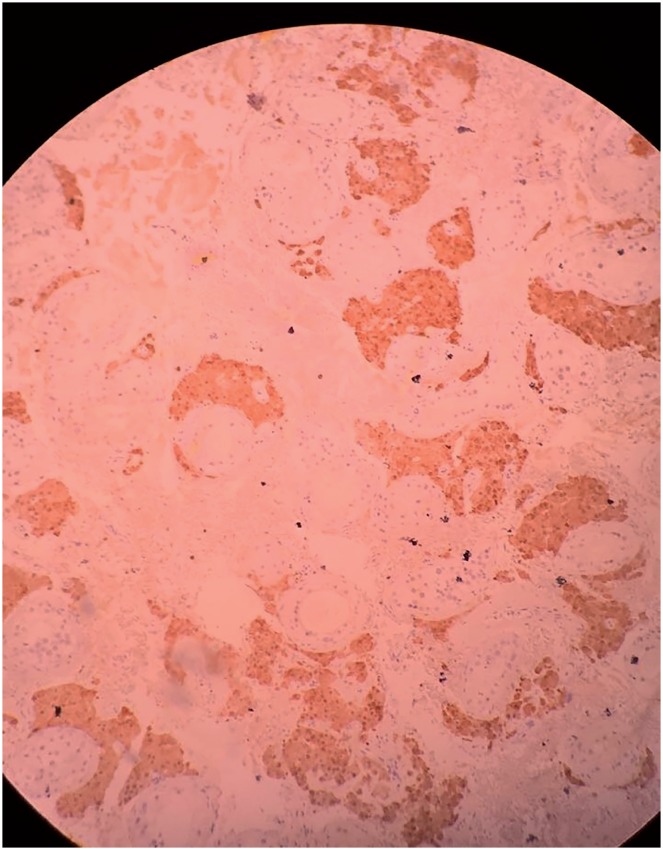
Seminiferous tubules (4x).

Ultrasound of scrotum is very useful to confirm the diagnosis of testicular tumor, but it cannot differentiate between a benign and a malignant tumor (10).

**Figure 3:**
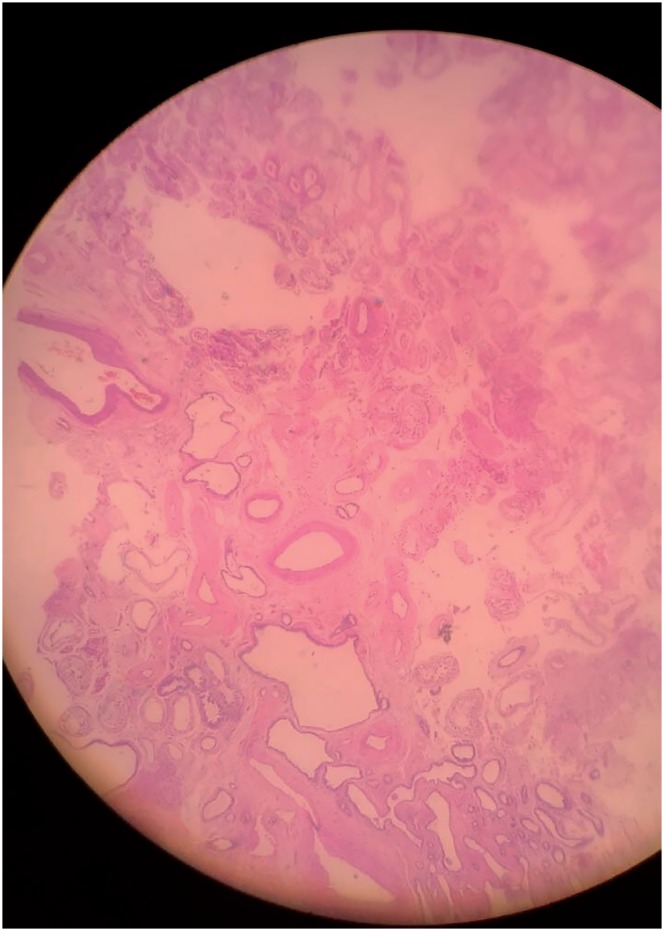
Rete Testis and Seminiferous tubules (4x).

**Figure 4:**
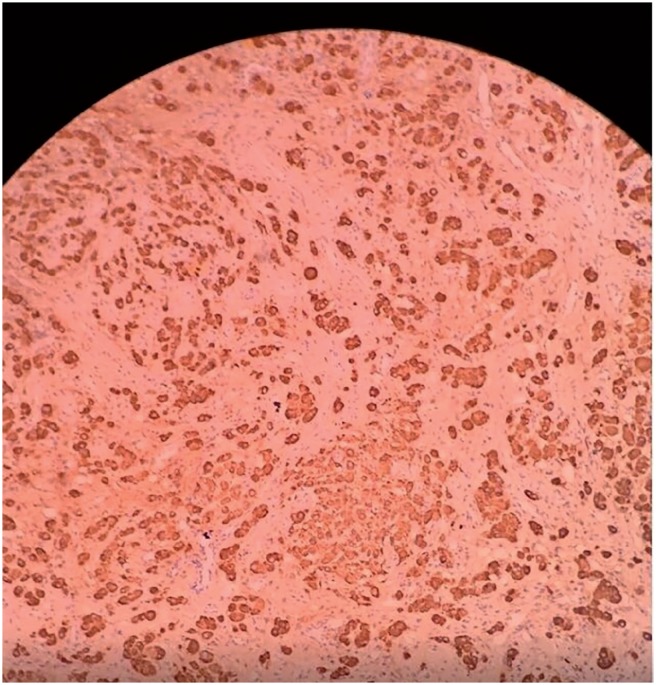
Inhibin antibody (4x).

**Figure 5:**
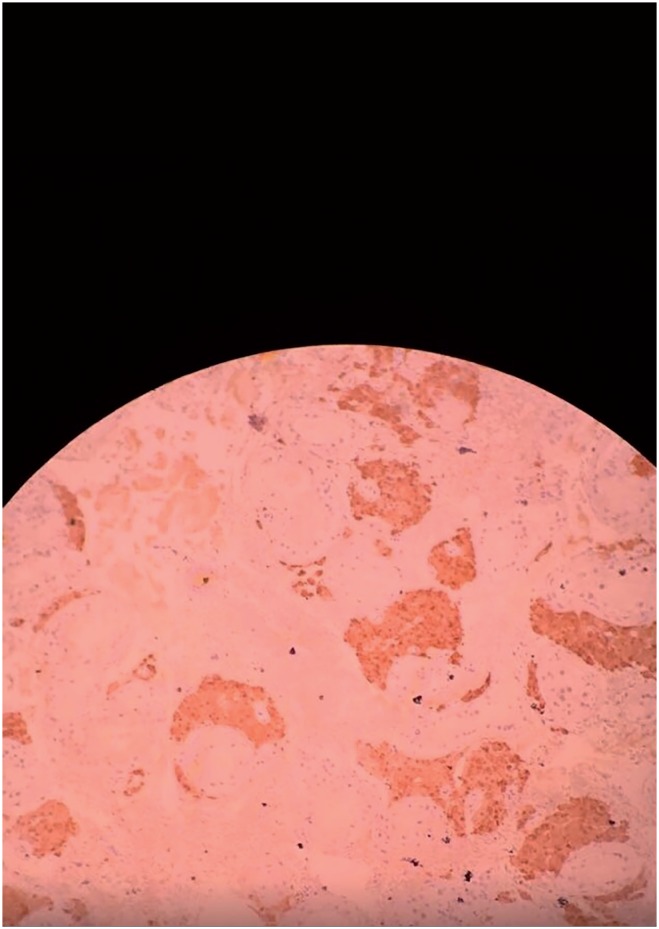
Calretinin antibody (4x).

**Figure 6:**
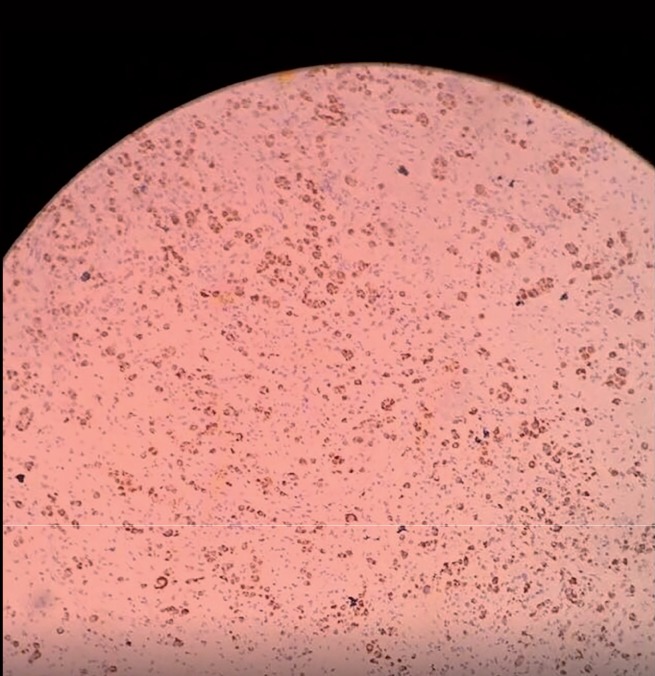
Melan-A antibody (4x).

**Figure 7:**
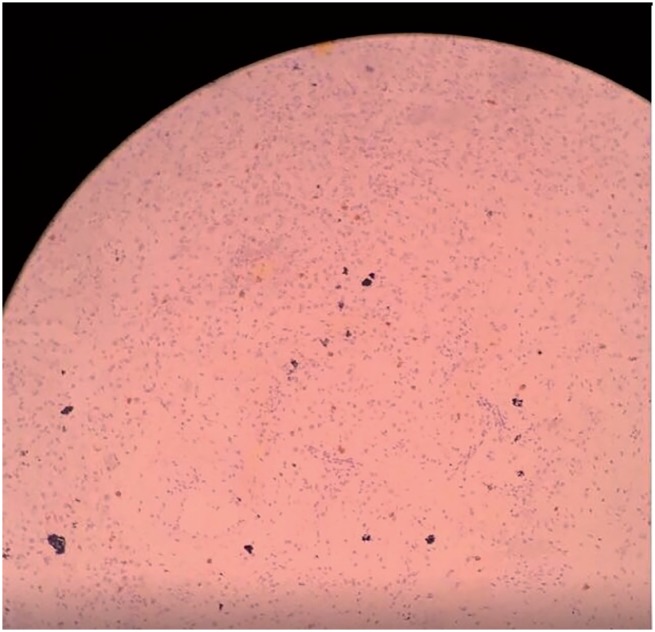
Ki-67 antibody (4x).

**Figure 8:**
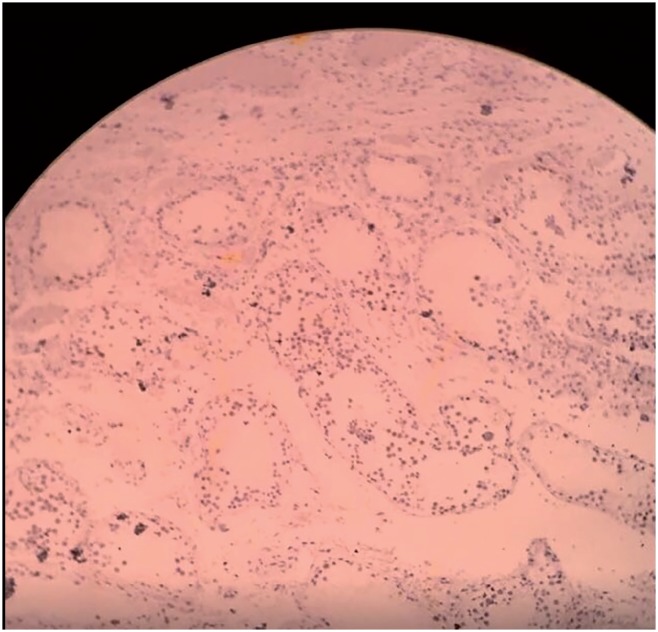
CD117 antibody (4x).

In case of infertility, gynecomastia, decreased libido or precocious puberty, luteinizing hormone (LH), follicle-stimulating hormone (FSH), testosterone, estrogen and estradiol should be dosed (11).

It should be distinguished from Leydig cell hyperplasia, which leads to atrophic testis, adjacent to germ tumors. Cell infiltrates seminiferous tubules without displacing or obliterating it. Unlike the tumor, they have normal values of urinary 17-ketoesteroides.

Macroscopically, they appear as nodules with coloration between brown and yellow, well circumscribed, without areas of necrosis or hemorrhage.

Histologically, the cells are large and round or polygonal, have abundant eosinophilic granular cytoplasm with a central round nucleus. About 25 to 40% demonstrate Reinke crystals. Reinke crystals contain lipofuscin pigment and have rounded shape, crystal structure with diameters from 3 to 20μm (2).

Avoiding surgery should be considered if lesions are smaller than 3cm and there is histological confirmation by freezing biopsy.

Leydig cell dysfunction and hypogonadism may occur after orchiectomy, and 40% of patients may require testosterone supplementation.

More than 70% of testicular tumors are diagnosed in the initial phase; the remainder are already metastasized at the time of diagnosis. The most common metastatic sites are regional lymph nodes, lung, liver and bones (8). Thus, retroperitoneal lymph node dissection is acceptable in selected cases with adverse characteristics, despite high rates of progression observed in positive lymph nodes, suggesting that the dissection would only have role in the staging.

Metastatic tumors are resistant to chemotherapy and radiation therapy, with low survival rates.
